# Low melting oxide glasses prepared at a melt temperature of 500 °C

**DOI:** 10.1038/s41598-020-80424-9

**Published:** 2021-01-08

**Authors:** Hirokazu Masai, Toru Nishibe, Satoshi Yamamoto, Takaaki Niizuma, Naoyuki Kitamura, Tomoko Akai, Takahiro Ohkubo, Miki Yoshida

**Affiliations:** 1 National Institute of Advanced Industrial Science and Technology, 1-8-31 Midorigaoka, Ikeda, Osaka 563-8577 Japan; 2 Ishizuka Glass Co. Ltd., 1880 Kawai-cho, Iwakura, Aichi 482-8510 Japan; 3 Graduate School and Faculty of Engineering, Chiba University, 1-33, Yayoi-cho, Chiba, 263-8522 Japan

**Keywords:** Glasses, Structure of solids and liquids, Materials for devices

## Abstract

Transparent low-melting inorganic glass is an attractive industrial material based on its high thermal and light resistance compared with conventional engineering plastics. If the melting temperature of inorganic glass could be decreased, the doping of guest materials or compression moulding on the glass surface would be easier. Although phosphate glass is considered as a potential candidate because of its transparency in the visible region and low-melting behaviour, water durability often becomes a problem for implementation. Here, we prepared inorganic low-melting phosphate glass at a temperature of 500 °C via a melting and quenching methodology. It was found that tin-doped phosphate glasses exhibited higher thermal and light resistance properties than polycarbonates. Colourless transparent oxide glasses without organic components are capable of bringing about new possibilities for the application of inorganic glasses.

## Introduction

Low-melting glass represents a conventional category of glasses whose softening temperatures are below 600 °C^1^. For several decades, lead-containing glasses were used as low-melting glass^2, 3^ because they possessed high chemical durability, refractive index, and transparency in the visible region, as well as low-melting behaviours. However, based on recent environmental requirements, according to the Restriction of Hazardous Substances Directive, the use of lead for conventional commercial materials is strictly prohibited. Therefore, many researchers have been investigating lead-free low-melting glass alternatives^4–8^. Notably, lead is a unique element with many advantages, as mentioned above, and it is very difficult to substitute lead cations with those of glass. Therefore, a combination of several elements is often used to satisfy these physical parameters^8–11^. However, some characteristics of lead-containing glass are generally achieved by sacrificing several properties. For example, vanadium-containing low-melting glass has no transparent window in the visible region. Thus, it is onerous to fabricate lead-free low-melting transparent inorganic glasses.

Recently, three-dimensional printing technology has become a popular tool for the production of solid-state materials, and good formability at low melting temperatures has become a key application issue. In addition, to match the thermal properties between glass and its counterparts, the low-temperature process of glass has been comprehensively examined. Engineering plastics (EPs) have been presumed as inorganic low-melting glass alternatives. This group of plastic materials evince several advantages, such as various chemical compositions, low density, low working temperature, and better brittleness relative to inorganic glasses. Nonetheless, EPs are strictly limited based on the perspectives of thermal and light resistance. Among the many industrial applications of EPs, optical utilization through light-emitting diodes (LEDs) is envisaged to grow in importance in the near future. Recent developments in LED applications entail the fabrication of next-generation light sources with high power and shorter wavelength (high photon energies) qualities. Although several EPs have been reported for this application, the thermal and light resistance properties of organic-containing glass are inherently inferior to those of organic-free inorganic glasses. If an inorganic glass could be fabricated at a much lower temperature than that of conventional industrial glasses, a novel glass system, which could be adopted by many users without a specific electric furnace, would ensue.

Although a lower preparation temperature is industrially preferable, it often results in materials with a correspondingly low chemical durability. For example, if a low-melting inorganic oxide glass is fabricated below 400 °C, satisfying both the low preparation temperature and chemical durability requirements is expected to be very difficult. Therefore, as a target temperature herein, we choose 500 °C as the fabrication temperature for an inorganic glass that can possess both low-melting behaviour and chemical durability. Notably, the sol–gel process is suitable for glass preparation at a lower temperature than the conventional melt-quenching method^12–14^. However, because the sol–gel process generally requires a preparation temperature above 500 °C, it is essential to examine alternative preparation methods for lower temperature synthesis. Based on a previous report, we focussed on the acid–base reaction for the preparation of organic–inorganic hybrid glasses^15^. A key factor entails the use of H_3_PO_4_ as both the reactant and the solvent of the batch, which allows for the formation of metal cation–oxygen–phosphorus bonds during the heat treatment of H_3_PO_4_ and metal chlorides. This concept has been previously confirmed to be adaptable for the preparation of inorganic films^16^. However, the spontaneous generation of HCl from the starting materials damages the reaction system, thereby constituting a challenge. Considering the less toxic leaving groups, we assumed that oxides, alkoxides, hydroxides, and phosphates could be used as starting materials. Ehrt reported the preparation of transparent SnO–P_2_O_5_ glasses at different melting temperatures^17^. It was discovered that the OH-containing SnO–P_2_O_5_ glass could be prepared at 450 °C, although the water durability was poor, whereas the same glass prepared at 1200 °C exhibited good durability. The water durability of phosphate glass is often problematic for practical applications^18–20^. If we could tailor the phosphate glass network at a lower temperature range, the potential of inorganic glasses as functional industrial materials would ensue. Therefore, we focussed on the SnO–P_2_O_5_-based glass system as the main chemical composition of glass and set the target preparation temperature as 500 °C. Herein, we report the preparation and physical properties of lead-free inorganic glasses prepared at 500 °C via a liquid-phase reaction, and it is demonstrated that transparent phosphate glasses possess a better thermal and high-power light resistance than conventional EPs.

## Results and discussion

### Examination of the chemical composition of tin phosphate-based glasses

Firstly, we examined the chemical composition of the host phosphate glass suitable for melting at 500 °C. There is a conventional relationship between the melting point (*T*_m_) and glass transition temperature (*T*_g_), i.e., *T*_g_/*T*_m_ ~ 2/3^21^. A benchmark of *T*_g_ is, therefore, approximately 243 °C to attain melting at 500 °C. In this study, we focussed on the SnO–P_2_O_5_ glass system because it was reported that SnO–P_2_O_5_ glasses generally exhibited lower *T*_g_ values below 300 °C^4, 5, 17, 22–24^. It is important to maintain the Sn^2+^ states during melting at 500 °C because it is reported that the oxidation reaction of Sn^2+^ starts at approximately 450°C^25^.

**Figure 1:**
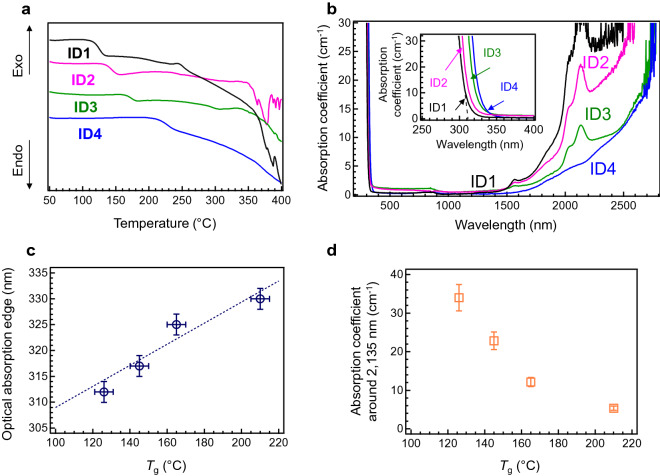
Thermal and optical properties of SnO–P_2_O_5_-based glasses: (**a**) DTA curves of the SnO–P_2_O_5_-based glasses listed in Table 1. (**b**) Optical absorption spectra of 50SnO–50P_2_O_5_ (ID1), 55.6SnO–44.4P_2_O_5_ (ID2), 60SnO–40P_2_O_5_ (ID3), and 10K_2_O–50SnO-40P_2_O_5_ (ID4) glasses melted at 500 °C for 30 min. Inset shows the expanded spectra at the optical absorption edge region. The dashed line indicates the ID1 extrapolation line for the optical absorption edge. (**c**) Relationship between the optical absorption edge and *T*_g_ of these glasses. (**d**) Relationship between absorption coefficient at approximately 2,135 nm and *T*_g_.

Table 1 presents the chemical compositions and *T*_g_ values of several SnO–P_2_O_5_-based glasses prepared at 500 °C. The differential thermal analysis (DTA) curves are shown in Fig. 1a. The error bars of these *T*_g_ values exceed 5 °C, which are marginally larger than the conventional error values estimated by extrapolating the DTA curve. An alternative composition of ID2 (50SnO–40P_2_O_5_) is also shown in order to understand the change in *T*_g_ with the addition of either 10 mol% of SnO (for ID3) or 10 mol% of K_2_O (for ID4). This suggests that the obtained glasses are thermodynamically metastable transient states whose concentration of OH groups^17^ or network formation is slightly different. Because the base chemical composition is similar, it is expected that a glass possessing a higher *T*_g_ exhibits a higher chemical durability. We occasionally observed a brownish colouration of the prepared glass in the 60SnO–40P_2_O_5_ glass, although all starting chemicals contained no carbon species. Since remarkable diffraction peaks are not observed in the brownish-coloured sample (Fig. S1), it is expected that small amounts of Sn nanocrystallites may be formed during melting. Figure 1b shows the optical absorption spectra of the 50SnO–50P_2_O_5_ (ID1), 55.6SnO–44.4P_2_O_5_ (ID2), 60SnO–40P_2_O_5_ (ID3), and 10K_2_O–50SnO–40P_2_O_5_ (ID4) glasses. The inset reveals the expanded spectra at the optical absorption edge region. If we evaluate the optical absorption edge from the extrapolation of the absorption coefficient, as shown by the dashed line in the figure, the compositional dependence can be elucidated. It was found that these optical absorption edges were located below 330 nm and that the optical absorption edges blue-shifted with decreasing *T*_g_ values, as shown in Fig. 1c. For the SnO-doped glass prepared by the conventional melt-quenching method, the optical absorption edge owing to the Sn^2+^ cation redshifts with increasing SnO fraction^26^. However, such an edge shift depending on the SnO fraction is not observed in the present case. Considering the *T*_g_, it is expected that the concentration of the OH group affects the blueshift of the optical absorption edge. This speculation is confirmed by the absorption spectra in the infrared (IR) region. The absorption coefficients of these glasses in the IR region increase with decreasing *T*_g_ values, thereby suggesting that a higher OH concentration induces a larger decrease in *T*_g_. The absorption bands at 1,570 nm and 2,135 nm are assigned to the overtone of the P–OH stretching and the combination stretching–bending of the P–OH modes, respectively^27, 28^. Figure 1d shows the absorption coefficient of the 2,135 nm peak as a function of *T*_g_. Notably, the surface of the 50SnO–50P_2_O_5_ glass with the highest OH concentration was rapidly damaged by water immersion at room temperature (RT). However, if the SnO fraction exceeded 60 mol%, the SnO–P_2_O_5_ glasses would need a melt temperature higher than 500 °C, and they sometimes exhibited opacity or a brownish colouration. The water durability of the ID1 glass was the worst, whereas ID4 was the best among these glasses. From these spectra, we concluded that ID4 (10K_2_O–50SnO–40P_2_O_5_) was the best candidate with both low-melting behaviour and chemical durability among these compositions. As the pH of water decreased (acidic) after the dissolution testing of these glasses, we can deduce that there is a conventional hydrolysis reaction mechanism between water and the phosphate chains^18^.

**Table 1:** Chemical compositions and the glass transition temperature (*T*_g_) values of SnO–P_2_O_5_-based glasses.

ID	Chemical composition (mol%)	*T*_g_ (°C)
ID1	50SnO–50P_2_O_5_	126
ID2	55.6SnO–44.4P_2_O_5_ [50SnO–40P_2_O_5_]	145
ID3	60SnO–40P_2_O_5_	165
ID4	10K_2_O–50SnO–40P_2_O_5_	210

**Figure 2:**
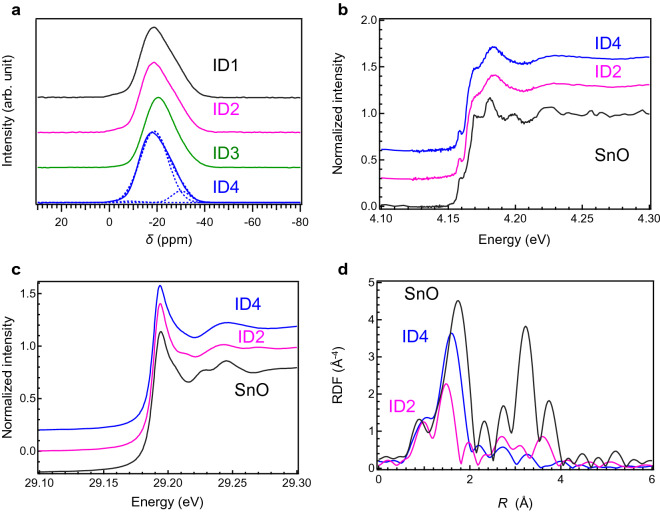
Structural analysis of SnO–P_2_O_5_-based glasses melted at 500 °C for 1 h. (**a**) ^31^P MAS NMR spectra of SnO–P_2_O_5_-based glasses. Dotted lines indicate Q^0^, Q^1^, and Q^2^ components after peak deconvolution. (**b**) Sn L_2_-edge XANES spectra of SnO–P_2_O_5_-based glasses along with that of SnO. (**c**) Sn K-edge XANES spectra of SnO–P_2_O_5_-based glasses. (**d**) FT of EXAFS of Sn K-edge XAFS. The *k* region for FT is from 3.4 to 12 Å^−1^.

We assumed that the water resistance of K_2_O–SnO–P_2_O_5_ glasses might depend on the structural change in the P_2_O_5_ region. To examine the structural changes based on the chemical composition, the ^31^P magic-angle spinning (MAS) nuclear magnetic resonance (NMR) spectra were measured. Figure 2a shows the ^31^P MAS NMR spectra of the SnO–P_2_O_5_-based glasses, which are listed in Table 2. The different phosphate units, Q^*i*^, in the ^31^P NMR spectra are identifiable from the chemical shift, which is attributed the number of bridging oxygen atoms^11, 31–33^. The peaks due to Q^2^, Q^1^, and Q^0^ in tin–phosphate-based glasses are located at − 33 ppm, − 19 ppm, and − 9 ppm, respectively^34^. It is evident from the NMR spectra that the dimer structure, Q^1^, whose chemical shift is approximately − 19 ppm, is the main phosphate unit in these glasses. The calculated fractions of Q^*n*^ units are shown in Table S1. In all the samples, a small amount of the Q^0^ unit was observed. It is previously proposed that the Q^0^ and Q^1^ units, which are highly electron-delocalized units, affect the chemical (water) durability compared to the Q^2^ unit. Put differently, if the fractions of such highly electron-delocalized units are large, the glass would exhibit excellent water durability^33^. However, contrary to our expectations, there was no remarkable difference between these glasses. In addition, although the fractions of (Q^0^ + Q^1^) exceed 80% in all the systems, these glasses have low water durability. Thus, we assume that the residual OH groups affect the water durability of these glasses because of the low melt temperature.

**Table 2:** Chemical compositions and weight loss of KSP glasses after the immersion test at 50 °C for 72 h. However, the 1.0La_2_O_3_-doped KSP glass was not obtained because of strong bubbling during the heat treatment at 500 °C.

Additive component (mol%)	Starting chemical	Weight loss (10^–6^ g/mm^2^)
−	−	22
0.5La_2_O_3_	La_2_O_3_	1.5
1.0La_2_O_3_	La_2_O_3_	−
0.5SiO_2_	Si(OC_2_H_5_)_4_	27
1.0SiO_2_	Colloidal silica	26
0.5Al_2_O_3_	Al(OH)_3_	45
1.0Al_2_O_3_	Al(OH)_3_	38
0.5B_2_O_3_	B(OH)_3_	23
1.0B_2_O_3_	B(OH)_3_	12

Since this preparation was conducted under ambient or reduced pressure conditions at 500 °C, which was comparable to the conventional temperature for Sn^2+^ oxidation, it was envisaged that most of the tin species were divalent^25^. Since Sn^2+^ is more preferable for low-melting relative to Sn^4+^, experimental confirmation is important^29^. To confirm the valence state of tin, we measured the Sn L-edge X-ray absorption near-edge structure (XANES) spectra according to a previous paper^29^. Figure 2b shows the Sn L_2_-edge of the ID2 and ID4 glasses, along with that of SnO. Compared with the reference (SnO), it is evident that approximately all Sn species are divalent, and the local coordination state of SnO does not drastically change with K_2_O addition. Because it was also envisaged that SnO connectivity would be affected by the chemical composition, we also measured the Sn K-edge X-ray absorption fine structure (XAFS) spectra. Figure 2c shows the Sn K-edge XAFS spectra of the ID2 and ID4 glasses with SnO as a reference. Because the XANES spectra shapes are similar, the result is consistent with valence estimation from the L_2_-edge. Figure 2d shows the Fourier transform (FT) of the extended XAFS (EXAFS) region of the Sn K-edge XAFS. The FT was performed with the *k* region from 3.4 to 12 Å^−1^. The addition of K_2_O changes the SnO structure, i.e., the Sn–O distance becomes longer, and the coordination number increases through K_2_O addition. It seems rather peculiar that the Sn–O distance of K_2_O-substituted SnO–P_2_O_5_ glasses is closer to SnO than that of the SnO-rich SnO–P_2_O_5_ glass. It has been reported that SnO has a tetragonal unit cell with a litharge structure^30^. However, it is speculated that the P=O bond of the P_2_O_5_ unit in the SnO–P_2_O_5_ structure can expand the interatomic distance of Sn^2+^ owing to the repulsion of electrons. It is, therefore, expected that potassium cations will be located near the phosphate chains for preferential charge compensation, and the residual Sn^2+^ cations will exist in a higher coordination state, similar to the SnO structure. Since the ^31^P NMR and Sn L_2_ edge XANES spectra are similar, the change in Sn cation coordination by the addition of K_2_O is one of the reasons for obtaining better water durability. Based on the results of the compositional survey, we selected 10K_2_O–50SnO–40P_2_O_5_ glass, which is hereafter denoted as KSP glass and possesses the lowest OH concentration and the highest *T*_g_ among these glasses, as the tin phosphate-based glass composition.

### Improvement of water durability of KSP-based glasses

**Figure 3:**
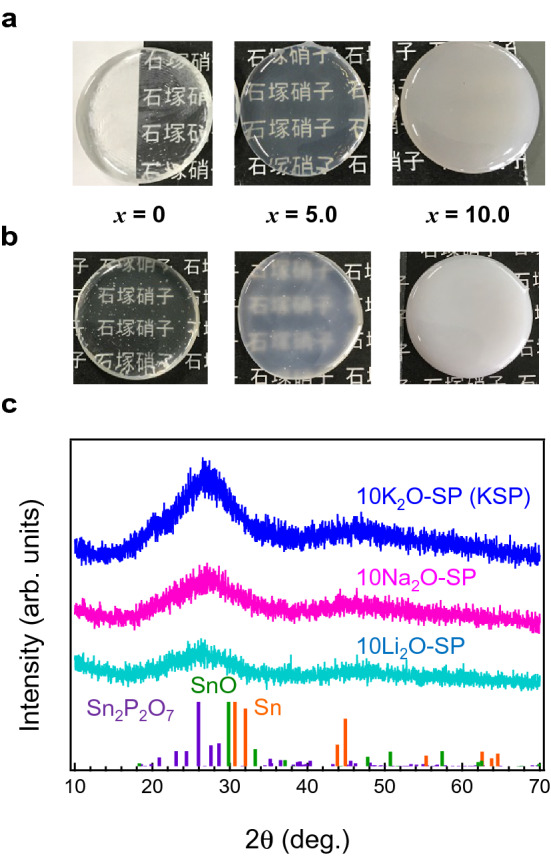
Transparency of alkaline-substituted KSP glasses. (**a**) Photographs of *x*Li_2_O − (10 − *x*)KSP and (**b)** *x*Na_2_O-(10-*x*)KSP glasses melted at 500 °C for 10 min. The substitution fractions are 0, 5, and 10. (**c**) XRD patterns of KSP, 10Na_2_O-SP, and 10Na_2_O-SP glasses along with JCPDS patterns of Sn (#00-004-0673), SnO (#01-072-1012), and Sn_2_P_2_O_7_ (#00-056-0358).

Although we select KSP glass as the main host composition, its water durability property is insufficient. Therefore, additional material design under a melting condition of 500 °C is needed. The dissolution behaviours of phosphate glasses are often discussed based on the nature of the glass surface and the P–O–P hydrolysis rates. It is natural that the composition and structure of glasses affect the dissolution behaviour. Alkali oxides and Q^2^ and Q^3^ phosphate units increase their dissolution rates, whereas high field strength cations, such as Al_2_O_3_ and Fe_2_O_3_, decrease the dissolution rates. It has occasionally been reported that the mixing of alkaline metal oxides, i.e., the mixed alkali effect of oxide glasses*,* improves the water durability of glasses^35, 36^. Recently, Onodera et al*.* proposed that the correlated pair arrangement of Na and K was the intrinsic origin of the mixed alkali effect. Based on previous reports, several glasses containing two alkaline metal oxides were prepared. Figure 3a shows a photograph of *x*Li_2_O − (10 − *x*)KSP glasses prepared at 500 °C. The transparency decreases with increasing Li_2_O concentration, and, in particular, the K_2_O-free glass is opaque, which is unsuitable for optical applications (see Fig. S2). A similar result was also confirmed in the Na_2_O-substituted *x*Na_2_O − (10 − *x*)KSP system (Fig. 3b). In the Na-substituted system, KSP glass also exhibited the best transparency among them. The crystallization behaviours are evaluated by acquiring XRD patterns. Figure 3c shows XRD patterns of KSP, 10Na_2_O-SP, and 10Na_2_O-SP glasses along with the Joint Committee on Powder Diffraction Standards (JCPDS) patterns of Sn (#00-004-0673), SnO (#01-072-1012), and Sn_2_P_2_O_7_ (#00-056-0358). In these glasses remarkable precipitation of crystallites was not observed. Therefore, it is envisaged that 500 °C is too low to make homogeneous glass melt to exhibit the mixed alkali effect, and only phase separation occurs during the melting. Although the reason K_2_O introduction presents the best outcome among the alkaline metal oxides remains unclear, it is presumed that the cationic radius of K_2_O is suitable for transparency and low-melting behaviour.

To improve the water durability of the low-melting KSP glass, we tried to add a fourth component. Table 2 shows the chemical compositions and weight loss of KSP glasses after the water immersion test at 50 °C for 72 h. The data in Table 2 show that the additive concentration seems to be less effective relative to the elements. Among several metal oxides, we discovered that La_2_O_3_ could effectively improve the water durability of the materials. An improvement of water durability through the addition of La_2_O_3_ has been reported in several glass systems prepared via the melt-quenching method^37, 38^. In the present glass system, improved water durability through La_2_O_3_ addition is also confirmed, even with a melt temperature of 500 °C, which is much lower than the conventional melt temperature. It is also notable that the addition of La_2_O_3_ induces no degradation on the transparency of KSP glass. We assume that La cations connect to phosphate units to prevent phase separation.

### Physical properties of La_2_O_3_-doped KSP (LKSP) glasses

**Figure 4:**
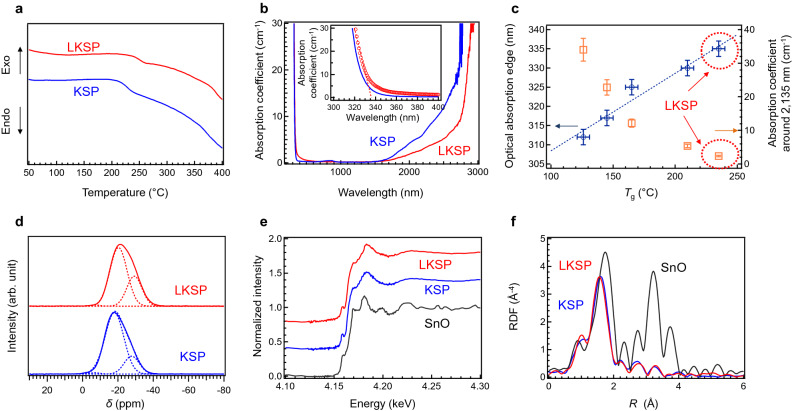
Effect of La_2_O_3_ addition on KSP glasses. (**a)** DTA curves of KSP glass and La_2_O_3_-doped KSP (LKSP) glass. (**b**) Optical absorption spectra of KSP and LKSP glasses. Inset shows the expanded absorption spectra at the absorption edge region. (**c**) Optical absorption edge and the absorption coefficient around 2,135 nm as a function of *T*_g_. (**d**) ^31^P MAS NMR spectra of KSP and LKSP glasses. (**e**) Sn K-edge XANES spectra of KSP and LKSP glasses along with SnO. (**f**) FT of EXAFS of Sn K-edge XAFS spectra of KSP and LKSP glasses along with SnO.

Previous data suggest that 0.5La_2_O_3_-doped KSP (LKSP) glass is a promising candidate for inorganic low-melting glasses. Over a course of three years, we confirmed that the LKSP glass is stable under ambient conditions (25 °C, ~ 60% humidity) despite the considerable amount of K_2_O, because La_2_O_3_ and SnO prevent the hydrolysis reaction. Therefore, we examined the structure and physical properties of the LKSP glass. Figure 4 presents a comparison between the KSP and the LKSP glasses. An increase in *T*_g_ upon La_2_O_3_ addition is observed, as shown in Fig. 4a, and simultaneously, a decrease in the OH concentration is apparent in the optical absorption spectra (Fig. 4b). Therefore, the decrease of OH groups by the addition of La_2_O_3_ is one of the origins of increasing *T*_g_. The effect of La_2_O_3_ addition is also observable at the optical absorption edge. Although the molar fraction of SnO slightly decreases with the addition of La_2_O_3_, the absorption edge redshifts, as shown in the inset of Fig. 4b. The relationship between *T*_g_ and these absorption properties was also confirmed in the La-doped sample, as shown in Fig. 4c. It is notable that the Q^*n*^ fractions in the ^31^P MAS NMR spectra are slightly changed by La_2_O_3_ addition (Fig. 4d), and the fraction of Q^2^ increases by addition of La_2_O_3_ (Table S1). In the case of iron-containing glass, it is proposed that added Fe cations connected to phosphate chains to improve the chemical durability^39, 40^. From the results of Q^2^ ratio, it is expected that La cations work the similar role to the previous report on the phosphate network^39^. Since a remarkable difference is not observed in the Sn L_2_-edge XANES (Fig. 4e) and Sn K-edge XAFS spectra (Fig. 4f), it is suggested that La cations mainly interact with phosphate units to decrease OH concentration at 500 °C heating, thereby improving the water durability of the LKSP glass.

Figure S3 shows the viscosity curve of an LKSP glass. The experimental data can be fitted using the Vogel–Fulcher–Tamman (VFT) equation^41–43^. From the viscosity curve, we can determine several thermal parameters, which are listed in Table 3. The thermal expansion coefficient and elastic parameters are also shown therein. From the thermal parameters, we can understand that the obtained glass can exhibit a low-melting behaviour, whose *T*_g_ is comparable to that of conventional low-melting glass^2–10^. The *T*_g_ of 235 °C is close to the expected *T*_g_ value (243 °C), based on the relationship: *T*_g_/*T*_m_ ~ 2/3^21^. Based on the fragility of glass-forming liquid defined by Novikov et al.^44^, the ratio of longitudinal and transversal sound velocity *v*_L_/*v*_T_ is approximately 1.76, indicating that the glass is strong rather than fragile. It is expected that surface tailoring via the nanoprinting method can be adapted to this glass below 400 °C, which is an advantage not only for decreasing temperature but also for the possibility of applying other types of moulds for superficial nanoprinting at the surface. Nonetheless, the refractive index at 633 nm of the LKSP glass is 1.65, which is higher than that of conventional EPs, thereby presenting another advantage of this low-melting inorganic glass.

**Table 3:** Thermal and elastic properties of the LKSP glass.

Glass transition temperature	235 °C
Density	3.40 g/cm^3^
Yield point	260 °C
Softening point (10^7.65^ poise)	320 °C
Working point (10^4^ poise)	420 °C
Thermal expansion coefficient (50–150 °C)	1.70 × 10^–5^/°C
Longitudinal sound velocity *v*_L_	3.48 km/s
Transverse sound velocity *v*_T_	1.98 km/s
Shear modulus	13.4 GPa
Poisson ratio	0.26
Young modulus	33.7 GPa
Bulk modulus	23.4 GPa

It is notable that the KLSP glass is hard to be prepared by conventional melting using Pt crucibles. As shown in Fig. S4, significant damage of surface of Pt crucible was detected after melting at 800 °C in Ar atmosphere. Therefore, the low-melting process at 500 °C using aqueous H_3_PO_4_ has additional advantage that damage of crucibles due to strong reduction reaction is prevented.

**Figure 5:**
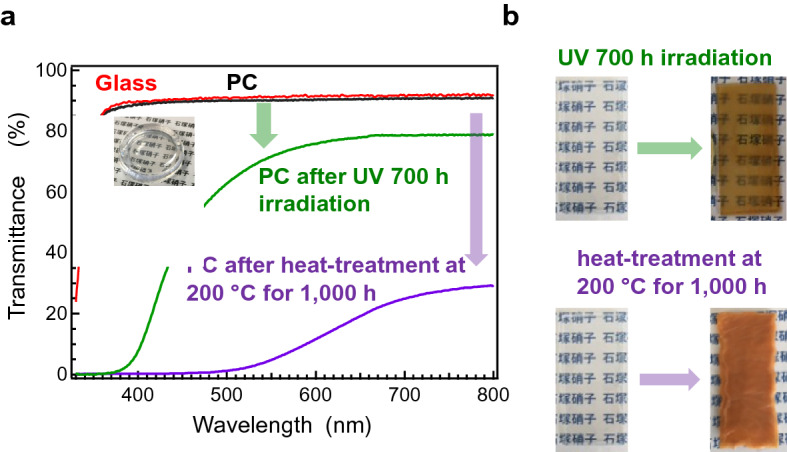
Transmittance of LKSP glass accelerated durability test by comparison with polycarbonates (PCs). (**a**) Transmittance spectra of LKSP glass and PCs after UV irradiation and heat treatment at 200 °C for 1000 h. (**b**) Photographs of PCs before and after durability tests. Remarkable transmittance degradation is observed in PCs, while no change is observed in the LKSP glass.

As mentioned in the Introduction, we assume that the counterpart of low-melting inorganic glass is EPs. Here, we compare the thermal and light resistance of the LKSP glass with those of conventional EPs and polycarbonates (PCs). Figure 5a shows the transmittance spectra of La_2_O_3_-doped KSP glass, as well as those of PCs after the accelerated durability test. For comparison, the thicknesses of PCs and glass were normalised to 1 mm. To check the properties, two accelerated tests were performed: (1) ultraviolet (UV) exposure at RT for 700 h and (2) heat treatment at 200 °C for 1000 h in an ambient atmosphere. The transparency of the inorganic glass remained unchanged after both durability tests, while a large degradation in PC transparency was observed after both tests. Figure 5b shows photographs of PCs before and after the UV exposure and thermal treatment durability tests. The transmittance of the PCs deteriorates drastically after both durability tests. Conversely, the transparency of the glass was unchanged after both accelerated durability tests. The results herein demonstrate that the present inorganic glass can be used in some application regions of EPs. Recently, inorganic glasses have been substituted by organic resins or EPs owing to their density, preparation cost, and preparation temperatures. However, from the viewpoint of thermal and high-power light resistance, advantages exist in employing inorganic glasses. By decreasing the preparation temperature, we have emphasised that several application windows are now open to inorganic glasses.

Summarily, we fabricated low-melting phosphate glass with a melt temperature of 500 °C. By selecting the chemical composition and the starting materials, colourless, transparent glasses can be obtained. The *T*_g_ is below 250 °C, which is a great advantage for melting and shaping at low temperatures. Although water durability occasionally constitutes a major problem for low-melting phosphate glass, this property can be improved by the composition and preparation process. Because this low-melting glass is durable against thermal and strong light conditions, it is considered as a novel colourless solid-state matter candidate that can partially function as a substitute for conventional EPs.

## Experimental

The starting materials for the low-melting glasses were H_3_PO_4_ (85%), SnO, as well as metal phosphate, such as KPO_3_, K_2_HPO_4_, and other metal oxides. These chemicals were mixed in a 60 mL cylindrical borosilicate glass beaker (inner diameter: φ50 mm, height: 55 mm, wall thickness: 1.3 mm, supplied by the Maruemu Corporation, Japan) at room temperature (RT). The mixture in the glass beaker was initially preheated to reduce residual concentrations of OH groups and H_2_O at approximately 400 °C. After the pre-heat treatment, the obtained solid was melted at 500 °C for 15–30 min in an ambient or reduced atmosphere. If the generation of bubbles was significant during melting, the pressure was manually reduced in the pressure range from 0.1 MPa to 1 kPa. The temperature whereat the inorganic glass is held in a molten state is referred to as the ‘melt temperature’ herein. The obtained glass was gradually cooled down to RT without temperature control or quenched at RT.

The glass transition temperature was determined through differential thermal analysis at a heating rate of 10 °C/min. To examine the crystalline phases in the prepared glass, we performed X-ray diffraction measurements using a RINT2100 (Rigaku, Japan) with Cu Kα radiation. The densities of the glasses were determined according to Archimedes’ method using distilled water as an immersion liquid. The refractive index was measured with a prism coupling method using a prism coupler with 473-, 633-, and 1,319-nm light sources (Metricon, USA). The elastic properties were measured by means of the ultrasonic pulse echo method using a DPR-300 (JSR ultrasonics, USA). The frequencies of the longitudinal and transverse waves were 10 MHz and 5 MHz, respectively. The viscosity curve was obtained through a viscometer with 10 mmΦ parallel plates using PRVM-1100 (Motoyama, Japan). Optical transmission spectra were measured using a UH4150 spectrophotometer (Hitachi High-Tech Science, Japan).

Furthermore, ^31^P MAS nuclear magnetic resonance (NMR) spectra of the glasses were acquired on a DELTA 600 spectrometer (JEOL, Japan) and a CMX-400 NMR spectrometer (JEOL, Japan) at a frequency of 161.80 MHz (CMX-400) and 242.95 MHz (DELTA 600), as well as a spin rate and relaxation delay of 10 kHz and 5 s, respectively. The chemical shifts were estimated with respect to an 85% H_3_PO_4_ aqueous solution (0 ppm).

The Sn K-edge X-ray absorption near edge structure (XANES) spectra were measured using the transmittance method, whereas the Sn L_2_-edge XANES spectra were evaluated via the fluorescent method. These measurements were performed at the BL14B2 beamline of SPring-8. The operated storage ring energy was 8 GeV with a conventional current of 100 mA. For the transmittance method, the samples were mixed with boron nitride and pelletised.

The water durability of the samples was tested through weight loss. The samples prepared via melt-quenching were cast into a stainless-steel cell. These glasses were then re-melted in a 25 mL cylindrical borosilicate glass beaker (inner diameter: φ35 mm, height: 45 mm, wall thickness: 1.3 mm, supplied by the Maruemu Corporation, Japan) to obtain the free surface. For the dissolution experiment, 10 mL of ultrapure water was added to the beaker. After heat treatment at 50 °C for 72 h, the samples were dried at 80 °C for 3 h under vacuum conditions. By comparing the sample weight before and after water immersion, the weight losses of each samples were calculated. The error bars of the weight loss were estimated to be ± 10 wt%.

## Supplementary Information

Supplementary information.
