# Publisher Correction: Low melting oxide glasses prepared at a melt temperature of 500 °C

**DOI:** 10.1038/s41598-021-89259-4

**Published:** 2021-04-30

**Authors:** Hirokazu Masai, Toru Nishibe, Satoshi Yamamoto, Takaaki Niizuma, Naoyuki Kitamura, Tomoko Akai, Takahiro Ohkubo, Miki Yoshida

**Affiliations:** 1 National Institute of Advanced Industrial Science and Technology, 1‑8‑31 Midorigaoka, Ikeda, Osaka 563‑8577 Japan; 2 Ishizuka Glass Co. Ltd., 1880 Kawai‑cho, Iwakura, Aichi 482‑8510 Japan; 3 Graduate School and Faculty of Engineering, Chiba University, 1‑33, Yayoi‑cho, Chiba, 263‑8522 Japan

Correction to: *Scientific Reports* 10.1038/s41598-020-80424-9, published online 08 January 2021

This Article contains an error in Figure 5 where parts of panel (a) were erased due to a conversion issue.

The correct Figure 5 appears below as Figure 1.

**Figure 1:**
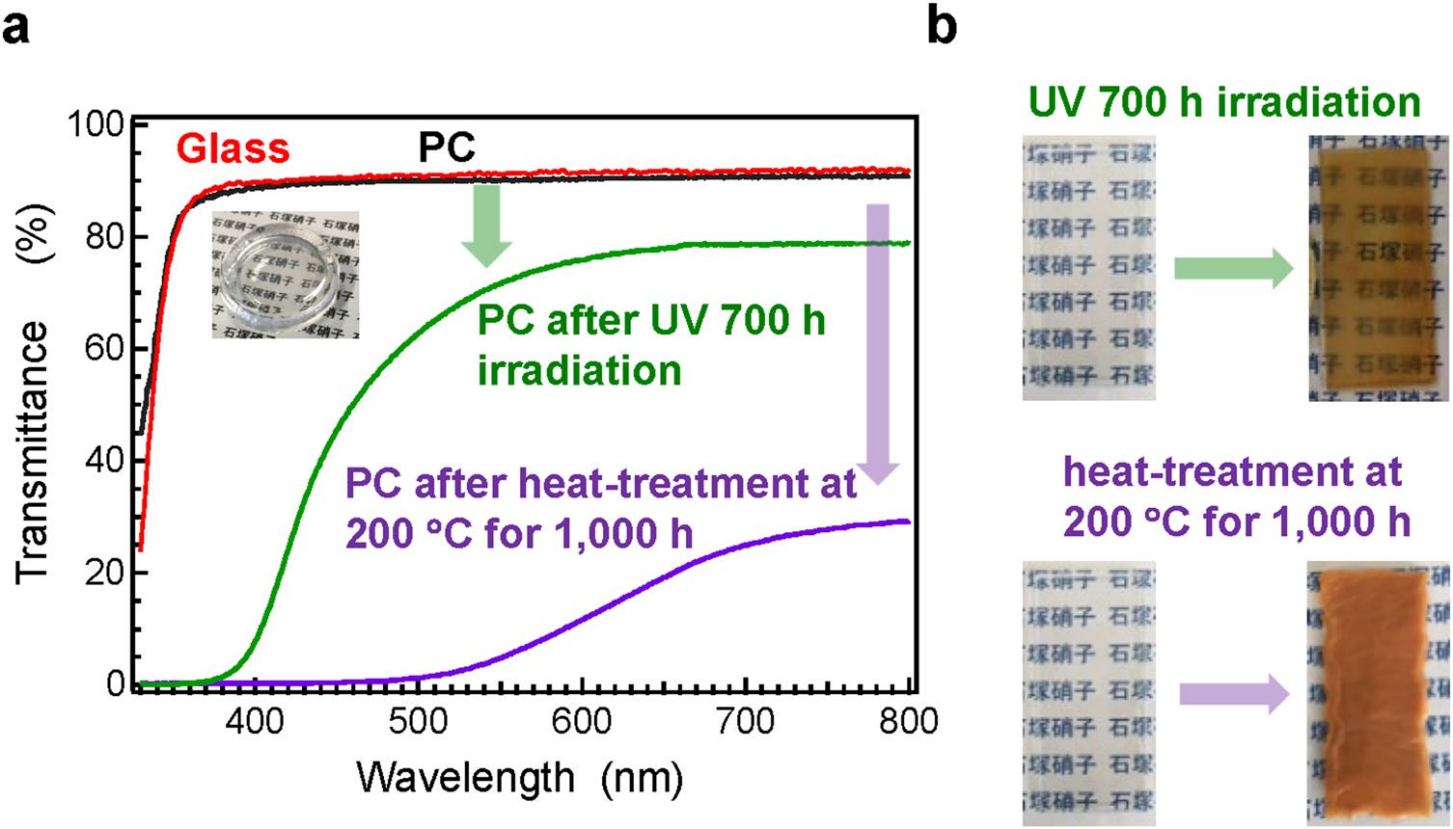
Transmittance of LKSP glass accelerated durability test by comparison with polycarbonates (PCs). (**a**) Transmittance spectra of LKSP glass and PCs after UV irradiation and heat treatment at 200 °C for 1000 h. (**b**) Photographs of PCs before and after durability tests. Remarkable transmittance degradation is observed in PCs, while no change is observed in the LKSP glass.

