# Supplementary material for: Low melting oxide glasses prepared at a melt temperature of 500 °C
Source: Sci Rep. 2021 Jan 8;11:214. doi: 10.1038/s41598-020-80424-9 (PMC7794583; doi:10.1038/s41598-020-80424-9)
Supplement: Supplementary file 1 — Supplementary information. [file 41598_2020_80424_MOESM1_ESM.docx]

*Supplemental information*

Low melting oxide glasses prepared at a melt temperature of 500 °C

H. Masai, T. Nishibe, S. Yamamoto, T. Niizuma, N. Kitamura, T. Akai, T. Ohkubo, and M. Yoshida

**Figure S1.**XRD pattern of brownish-coloured 60SnO–40P_2_O_5_-based glasses prepared at 500 °C. The JCPDS patterns of Sn, SnO, and Sn_2_P_2_O_7_ are also shown for comparison.

**Table S1.** Chemical compositions and fractions of phosphate units calculated from ^31^P MASNMR spectra.

| ID | Q^0^ (%) | Q^1^ (%) | Q^2^ (%) | Q^0^ + Q^1^ (%) |
| --- | --- | --- | --- | --- |
| ID1 | 3.3 | 78.5 | 18.2 | 81.8 |
| ID2 | 3.3 | 77.7 | 19.0 | 81 |
| ID3 | 1.7 | 80.0 | 18.2 | 81.7 |
| ID4 (KSP) | 0.9 | 80.3 | 18.8 | 81.2 |
| LKSP | 0.8 | 68.5 | 30.7 | 69.3 |

**Figure S2**

**(a)** Photographs of *x*Li_2_O-(10-*x*)KSP and **(b)** *x*Na_2_O-(10-*x*)KSP glasses melted at 500 °C for 10 min. The substitution fractions are 0, 2.5, 5, 7.5, and 10.

**Figure S3**. **Viscosity of the LKSP glass as a function of temperature.** The dotted and solid lines represent the experimental data and fitting curve, respectively, using VFT equation. The open circle and triangle indicate the softening and working points, respectively.

**
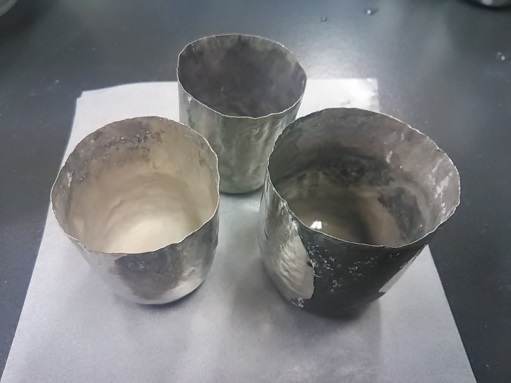
**

Formation of Sn-Pt alloys

**Figure S4.**A photograph of Pt crucibles after melting LKSP glass at 800 °C in Ar atmosphere. Starting materials were La_2_O_3_, KPO_3_, SnO, and NH_4_H_2_PO_4_.
